# Candidate Animal Disease Model of *Elizabethkingia* Spp. Infection in Humans, Based on the Systematic Pathology and Oxidative Damage Caused by *E. miricola* in *Pelophylax nigromaculatus*

**DOI:** 10.1155/2019/6407524

**Published:** 2019-09-18

**Authors:** Xiaoli Huang, Yang Feng, Hong Tang, Guanqing Xiong, Liangyu Li, Yucen Yang, Kaiyu Wang, Ping Ouyang, Yi Geng, Defang Chen, Shiyong Yang

**Affiliations:** ^1^College of Animal Science and Technology, Sichuan Agricultural University, Chengdu, Sichuan 611130, China; ^2^Chengdu Academy of Agricultural and Forestry Sciences, Chengdu, Sichuan 611130, China; ^3^College of Veterinary Medicine, Sichuan Agricultural University, Chengdu, Sichuan 611130, China

## Abstract

Most species of the genus *Elizabethkingia* are pathogenic to humans and animals, most commonly causing meningitis. However, our understanding of the pathogenic mechanisms involved is poor and there have been few pathological studies of *Elizabethkingia* spp. in animals. To understand the host injury induced by *Elizabethkingia* spp., we established a model of *E. miricola* infection in the black-spotted frog (*Pelophylax nigromaculatus*). The systematic pathology in and oxidative damage in the infection model were investigated. Our results show that recently isolated *E. miricola* is a bacterium that mainly parasitizes the host brain and that neurogenic organs are the predominant sites of damage. Infection mainly manifested as severe brain abscesses, meningoencephalitis, necrotic spondylitis, and necrotic retinitis. The liver, spleen, kidney, gastrointestinal tract, and lung were also affected to varying degrees, with bacterial necrotic inflammation. *P. nigromaculatus* also suffered enormous damage to its oxidative system during *E. miricola* infection, which may have further aggravated its disease state. Our results provide a preliminary reference for the study and treatment of *Elizabethkingia* spp.*-*induced neurological diseases in animals.

## 1. Introduction

*Elizabethkingia* is a new genus of Gram-negative rod-shaped bacteria, described in 2005, that were initially classified in the genus *Chryseobacterium* [1]. The genus includes four species, *E. meningoseptica* [2], *E. miricola* [3], *E. anopheles* [4], and *E. endophytica* [5], and almost all these species are strongly pathogenic to both humans and other animals [6–8]. Cases of human infection with *Elizabethkingia* spp. have been reported in the United States [9], England [10], Italy [11], Saudi Arabia [6], China [12], Central Africa [7], and India [13]. The disease is fatal in more than 50% of cases and can lead to meningitis, brain abscess, and other severe postinfection sequelae, including hydrocephalus, deafness, and developmental delay [14]. According to incomplete statistics, the mortality rate among people infected with *Elizabethkingia* spp. is 65% according to the Centers for Disease Control and Prevention (https://www.cdc.gov/). Newborns and immunocompromised adults are most susceptible to infection with *Elizabethkingia* spp. [15–19] and are likely to suffer symptoms of irritability, poor feeding, seizure, cloudy cerebrospinal fluid, and polymorphonuclear pleocytosis [7]. These symptoms are often identified and treated as influenza or another disease, and this misdiagnosis, combined with the lack of an effective therapeutic regimen, makes patients almost impossible to cure after infection with *Elizabethkingia* spp., which ultimately leads to death. In general, meningoencephalitis is the commonest symptom of human *Elizabethkingia* spp. infection [6]. However, research into the characteristics and mechanisms of the damage caused by *Elizabethkingia* spp. in the host is extremely limited, as is the prevention and treatment of the associated diseases.

Interestingly, several studies have found that *Elizabethkingia* spp. can cause meningoencephalitis in frogs, including the African clawed frog (*Xenopus laevis*) [20], bullfrog (*Rana catesbeiana* Shaw) [21], leopard frog (*Lithobates pipiens*) [22], tiger frog (*Rana rugulosa*) [23], black-spotted frog (*Pelophylax nigromaculata*) [24], and spiny frog (*Quasipaa spinosa*) [25]. Infected frogs display severe neurological symptoms and meningoencephalitis similar to those in humans. Therefore, the frog may be a natural model organism for research into the meningoencephalitis induced by *Elizabethkingia* spp. in humans. Frogs also have the advantage of being more readily available for research than humans, which is an essential factor in the study of disease.

In August 2017, wryneck disease broke out on a black-spotted frog farm in Sichuan, China. We isolated three strains of bacteria from the diseased frogs and identified them as *E. miricola*. To systematically explore the mechanisms underlying the invasiveness and damage caused by *Elizabethkingia* spp. in animals, an *Elizabethkingia* spp.-induced black-spotted frog infection model was established based on the strains isolated in the present study. The systematic pathology, the parasitized and damaged target organ(s), and the oxidative damage caused by the bacterium were investigated in the model. Our findings extend our understanding of the meningoencephalitis induced by *Elizabethkingia* spp. in both aquaculture organisms and humans and provide a reference for its prevention and treatment.

## 2. Methods

### 2.1. Bacterial Strain and Characteristics

The pathogenic strain used in this study was originally isolated from *P. nigromaculatus* with wryneck disease on a frog farm in Chongzhou, Sichuan, China, in 2017. The pathogen was isolated from the kidneys, livers, spleens, and brains of the diseased frogs on brain heart infusion (BHI) medium, and the colonies were picked for further purification. The isolated strain was cultured at 25°C for 18–24 h.

The bacterial species was identified with standard biochemical assays (Tianhe, Hangzhou, China) and 16S rRNA sequencing (PCR primers: F, 5′-AGAGTTTGATCCTGGCTCAG-3′ and R, 5′-GGCTACCTTGTTACGACTT-3′). The amplified sequences were compared with the GenBank database (https://www.ncbi.nlm.nih.gov/) with a BLAST analysis. A homology analysis was performed with MEGA 7.0 and a phylogenetic analysis with the neighbor-joining method. The agar diffusion method (Kirby-Bauer method) was used to determine the sensitivity of the isolated strains to 11 antibiotics (Tianhe).

### 2.2. Animals

Three hundred healthy *P. nigromaculatus* (torso length 6 ± 1.2 cm, weight 35.5 ± 3.5 g) were purchased from a commercial frog farm (Meishan, Sichuan, China) and acclimated at our facility for 2 weeks. Healthy individuals were chosen for the experiment at the end of the acclimation period. The frogs were fed commercial pellets twice a day and maintained under a light : dark cycle of 12 : 12 h. The water temperature contained ammoniacal nitrogen and nitrite at 0–0.02 mg/L, at a temperature of 22 ± 2°C and pH of 7.0–7.5. The culture water was renewed every day, after pretreatment with an aeration process. All animal handling procedures were approved by the Animal Care and Use Committee of Sichuan Agricultural University (Chengdu, China) and followed the guidelines for animal experiments of Sichuan Agricultural University, under permit number 20181102002. All chemicals were of analytical grade or the highest grade available from local companies.

### 2.3. Establishment of the Meningitis Model

Responsive, robust, healthy frogs were selected for the experiment. The challenged frogs (*n* = 120) were divided into four groups—a control group (treated with 0.65% physiological saline) and three experimental groups: 1 × 10^8^ group (treated with 1 × 10^8^ colony-forming units (CFU)/mL purified strain), 1 × 10^9^ group (treated with 1 × 10^9^ CFU/mL purified strain), and 1 × 10^10^ group (treated with 1 × 10^10^ CFU/mL purified strains). Each group included three parallel tanks. The purified strain (1 × 10^8^, 1 × 10^9^, or 1 × 10^10^ CFU/mL) was injected intramuscularly into each frog in the experimental groups, and the frogs in the control group were injected with physiological saline. The entire experimental period was 14 days. All gross lesions and deaths in the frogs of each group were recorded daily, and the dead frogs were removed at that time. On day 14 after injection, the frogs in each group were euthanized with tricaine mesylate (MS-222) (Sigma-Aldrich, Beijing, China) and dissected for histopathological analysis and the analysis of viable counts, enzyme activity, and mRNA expression.

### 2.4. Detection of Target Organs with Major Lesions

Three frogs in each group were humanely euthanized with MS-222. The skin, muscles, eyes, lung, brain, heart, liver, spleen, and kidneys were removed, fixed in neutral-buffered formalin, and dehydrated in ethanol. The tissues were trimmed into cassettes, dehydrated in graded ethanol solutions, cleared in xylene, and embedded in paraffin wax. Sections (3 *μ*m) were prepared for hematoxylin and eosin staining before a microscopic analysis (Nikon, Tokyo, Japan).

The degrees of hemorrhage, edema, deposits, hypertrophy, hyperplasia, atrophy, infiltration, and necrosis in the organs were evaluated according to the scoring system designed by Bernet et al. (Table 1) [26]. Every change was assessed with a score (*S*) ranging from 0 to 6, depending on the degree and extent of the change: (0) unchanged, (2) mild change, (4) moderate change, and (6) severe change (diffuse lesion). Intermediate values were also considered. The organ index (*I* = ∑_*t*_∑_alt_[*S* × *ω*_IF_]) and total index (*I* = ∑_Org_∑_*t*_∑_alt_[*S* × *ω*_IF_]) of each experimental group were calculated in this study (*ω*_IF_: importance factor).

### 2.5. Ultrastructural Examination

The livers of three frogs from each group were sampled, immediately fixed in 2.5% glutaraldehyde, and postfixed in 2% veronal-acetate-buffered osmium tetroxide. After dehydration in graded alcohol, the samples were embedded in Araldite. The blocks were sectioned on a microtome with a glass knife. The sections (6.575 *μ*m thick) were placed on uncoated copper grids, stained with uranyl acetate, and poststained with 0.2% lead citrate. The subcellular structures of the liver were examined with a Hitachi H-600 Transmission Electron Microscope (Hitachi, Tokyo, Japan).

### 2.6. Bacterial Colonization of Target Organs

Specific primers were designed to bind the 16S rRNA sequence of *E. miricola* (F: 5′-CGAACTGCCATTGATACTG-3′ and R: 5′-CGCTTAGTCTCTGAATCCTA-3′). Bacteria were randomly isolated from frogs in the experimental groups and identified with PCR using the 16S-rRNA-specific primers. The PCR thermal cycling conditions were 3 min at 95°C followed by 30 cycles of 30 s at 95°C, 30 s at 60°C, and 20 s at 72°C. The PCR product was identified with 2% agarose electrophoresis (product size: 234 bp).

Three frogs in the 1 × 10^10^ group were euthanized, and their livers, spleens, brains, lungs, kidneys, hearts, and muscles were dissected in a sterile environment. The live bacteria in the tissues were quantified with the viable count method. Briefly, the tissues were weighed in a sterile environment, homogenized in physiological saline (1 : 9), serially diluted, and spread on BHI medium. The colonies that appeared after incubation for 24 h at 25°C were counted to calculate the number of viable cells in the tissues.

### 2.7. Determination of Antioxidant Indices

The livers were removed from three frogs in each group and immediately homogenized in physiological saline at 4°C. Each homogenate was centrifuged at 3500 × g at 4°C, and the supernatant was used to detect different indicators. The total protein in the supernatant was determined with a quantitative total protein assay kit (A045-2; NJJCBIO, Nanjing, China). The total antioxidant capacity (TAC), the activity of superoxide dismutase (SOD), and malondialdehyde (MDA) were detected with biochemical kits (NJJCBIO, Nanjing, China), following the manufacturer's protocol.

### 2.8. Gene Expression of Antioxidants

The livers were removed from three frogs in each group, immediately placed in RNAiso Plus (TaKaRa, Japan), and stored at −80°C. The livers were then homogenized by crushing them, and the total RNA was isolated with the Simply P Total RNA Extraction Kit (Bioflux, China), according to the instruction of the manufacturer. The total RNA (1 *μ*g) was converted to first-strand cDNA with the PrimeScript™ RT Reagent Kit with gDNA Eraser (RR047A, TaKaRa). Quantitative PCR (qPCR) was performed with the One Step SYBR® PrimeScript™ RT-PCR Kit II (Perfect Real Time) (TaKaRa) and a thermocycler (Bio-Rad, USA).

Gene expression was analyzed with quantitative real-time PCR (qPCR) which was conducted in a StepOnePlus Real-Time PCR System (Applied Biosystems, USA). Three antioxidation-related genes (superoxide dismutase (*SOD*), catalase (*CAT*), and glutathione peroxidase (*GPX*)) were detected with this assay, and the housekeeping genes encoding *ribosomal protein L8* (*RPL8*) and *18S rRNA* were used as the internal standards (Table 2) [27, 28]. The primers are shown in Table 2. For qPCR, the 10 *μ*L reaction mixture contained 5 *μ*L of SYBR Green II PCR Master Mix, 3 *μ*L of diethyl-pyrocarbonate-treated water, 0.4 *μ*L each of the forward and reverse primers (10 *μ*mol/L), 0.2 *μ*L of ROX reference dye (TaKaRa), and 1 *μ*L of cDNA (100 ng/*μ*L). The PCR conditions included initial denaturation for 3 min at 95°C, followed by 40 cycles of 95°C for 10 s, the melting temperature of the specific primer pairs for 30 s, 95°C for 10 s, and 72°C for 10 s. To distinguish between specific and nonspecific reaction products, a melting curve was constructed at the end of each run. The 2^−ΔΔCT^ method was used to calculate the relative changes in mRNA expression from the qPCR results (ΔCT = CT_target gene_ − CT_housekeeping gene_, ΔΔCT = ΔCT experimental − ΔCT control) [29].

### 2.9. Statistical Analysis

The results are expressed as means ± standard deviations. The significance of differences was determined with analysis of variance. Each indicator was tested with one-way analysis of variance and a *t*-test. However, prevalence and mortality were analyzed with the Kaplan-Meier method and a log-rank test to determine whether the differences between the groups were significant (SPSS v.20.0, IBM Corp., New York, NY, USA). A value of *P* < 0.05 was considered significant, and *P* < 0.01 was considered highly significant.

## 3. Results

### 3.1. Source and Identification of the Strains

**Figure 1:**
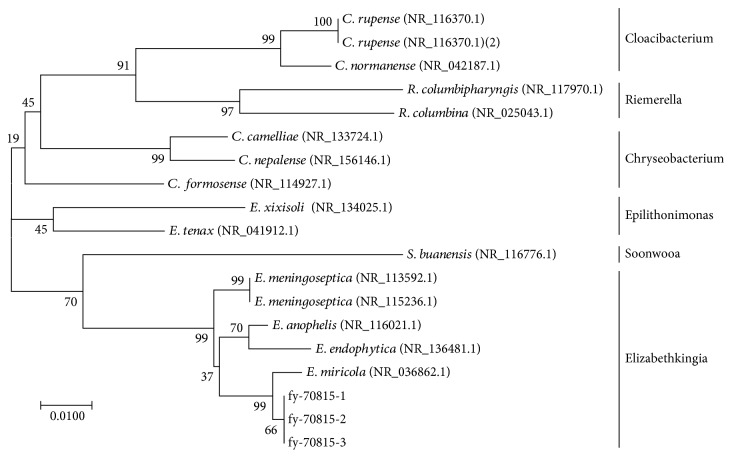
The phylogenetic tree was generated with MEGA 7.0 based on an alignment of the 16S rRNA gene sequences of the isolated strains (fy-70815 1–3) and related species.

The pathogenic strain was originally isolated from *P. nigromaculatus* with wryneck disease and identified. The colonies were round or elliptical, translucent, shiny, and smooth, with a diameter of 1–3 mm. Under a microscope (1000x), the bacteria presented as Gram-negative rods, singly or in pairs (0.5–1.0 *μ*m × 1.0–2.5 *μ*m). The biochemical characteristics of the isolates are shown in Table 3 and are consistent with those of the genus *Elizabethkingia*. The nearly full-length 16S rRNA gene sequences of the isolates were amplified, identified with a BLAST search, and deposited in GenBank (accession numbers: MK333252, MK333253, and MK333254). The three isolates shared the greatest nucleotide homology (99%–100%) with *E. miricola* (NR_036862.1) (Figure 1). The susceptibilities of the three isolates to antibiotics are shown in Table 4. They were all most sensitive to azithromycin, rifampicin, and florfenicol, followed by enrofloxacin, and were resistant to neomycin, doxycycline, penicillin, cotrimoxazole, cefoxitin, and ampicillin.

### 3.2. Gross Lesions in *E. miricola*-Infected *P. nigromaculatus*

**Figure 2:**
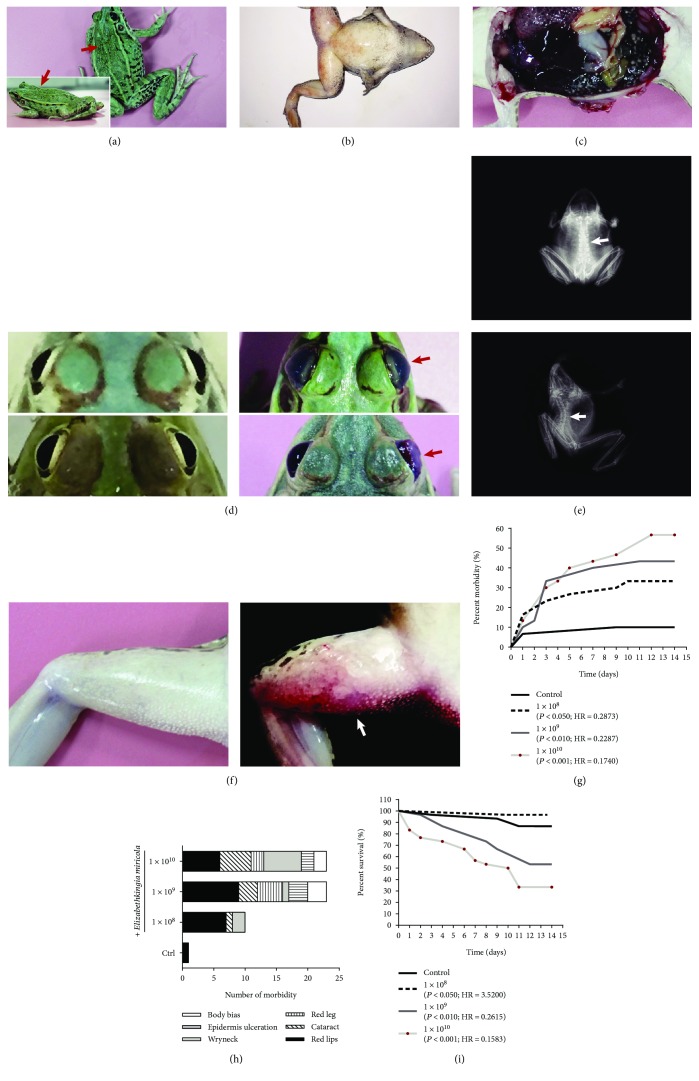
Gross lesions in *E. miricola*-infected *P. nigromaculatus*. (a) Head biased to one side; (b) abdomen and thigh swelling; (c) congestion of the lungs, hypoplasia of the ovary; (d) left: normal eyes and right: cataracts; (e) top: normal spine and bottom: rachiocampsis; (f) left: normal thigh and right: redness on frog thigh. (g) Morbidity (%) among *P. nigromaculatus* during *E. miricola* infection. (h) Symptom types in frogs of different groups. (i) Survival (%) of *P. nigromaculatus* during *E. miricola* infection. *P* values and hazard ratios (HRs) are relative to the control group.

Artificially infected *P. nigromaculatus* displayed symptoms similar to those of wryneck disease in nature. The commonest clinical signs in the sick frogs are side-biased swimming, with the gross lesions of wryneck, side-biased body (Figure 2(a)), edema of the abdomen and thigh (Figure 2(b)), cataract (Figure 2(d)), hemorrhage in the inner thigh skin (Figure 2(f)), congestion of the lung, and hypoplasia of the ovary (Figure 2(c)). X-rays also showed severe rachiocampsis in *P. nigromaculatus* after *E. miricola* infection (Figure 2(e)).

The prevalence and mortality of *E. miricola* infection in the frogs were both time and concentration dependent. As the period of challenge time and the challenge concentration increased, both the prevalence of infection and frog mortality increased. The morbidity rates were much higher in the 1 × 10^8^, 1 × 10^9^, and 1 × 10^10^ groups than in the control group, by 33.3%, 43.3%, and 56.7%, respectively. *Elizabethkingia miricola* also caused single or multiple concurrent symptoms in the different experimental groups of *P. nigromaculatus* (Figure 2(g)). Wryneck was not a common clinical symptom in the beginning of the disease process in the frogs. Low concentrations of *E. miricola* predominantly caused mouth redness and body bias, whereas high concentrations were mainly responsible for cataract and wryneck (Figure 2(h)). The mortality rates also differed across the experimental groups, and mortality was particularly evident in the high-concentration group. In the 1 × 10^10^ group, the bacterium caused death in nearly 70% (67.7%) of *P. nigromaculatus* and in nearly 50% (46.7%) of the 1 × 10^9^ group (Figure 2(i)).

### 3.3. Systematic Pathology in *E. miricola*-Infected *P. nigromaculatus*

**Figure 3:**
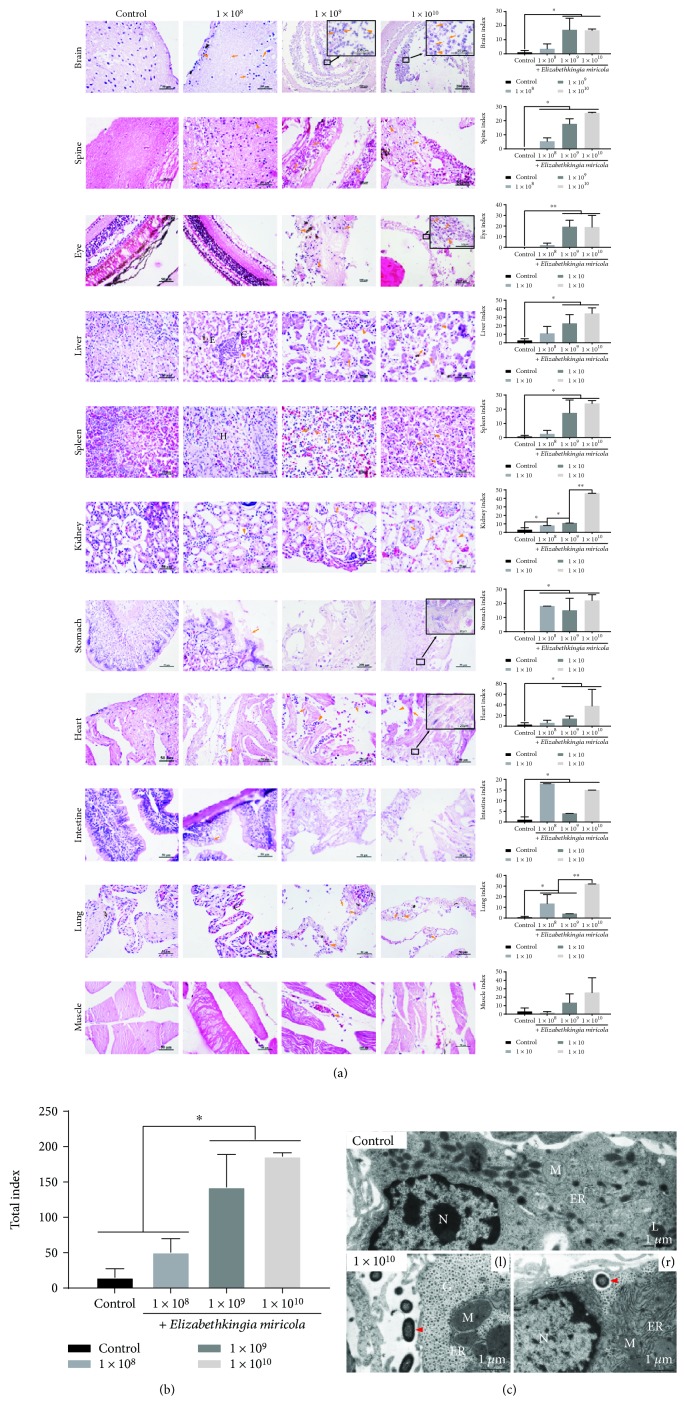
Dynamic systematic pathology of *P. nigromaculatus* caused by *E. miricola* infection. (a) Histopathological changes induced by *E. miricola* (▲: inflammatory cell infiltration; →: necrotic cells; E: edema; C: congestion; H: hyperplasia). (b) Overall health status (total index) of frogs in different groups, based on histopathological lesions. (c) Ultrastructural changes induced by *E. miricola* (N: nucleus; M: mitochondria; ER: endoplasmic reticulum; L: lysosome). *P* < 0.05 or ^∗∗^*P* < 0 01 represents a significant difference or highly significant difference between groups. Dynamic systematic pathology of *P. nigromaculatus* caused by *E. miricola* infection.

The frogs showed systemic histopathological changes depending on the concentration of *E. miricola* with which they were injected. The main target organs were the brain, spinal cord, and eyes. *Pelophylax nigromaculatus* in the 1 × 10^8^ group showed the earliest pathological changes, followed by the 1 × 10^9^ group and then the 1 × 10^10^ group, whereas *P. nigromaculatus* in the control group showed no obvious histopathological changes (Figure 3(b)). Severe pathological changes were observed in the low-concentration group (1 × 10^8^), which were mainly moderate to severe meningitis, spondylitis, and retinitis. The liver showed moderate edema and local inflammation, the spleen showed a reduction in interstitial cells and hyperplasia of the reticular system, and the kidney interstitium showed mild inflammation. Other organs were less affected by the low concentration of *E. miricola*, with only slight detachment of the gastric mucosal epithelium and mild inflammation in the cardiac chamber. As the concentration of injected bacteria increased (1 × 10^9^ and 1 × 10^10^), the extent of these lesions increased. They were mainly expressed as marked bacterial necrotic brain abscesses and meningoencephalitis, marked bacterial necrotic myelitis, severe retinal necrosis, necrotic hepatitis, necrotic spleen, or necrotic nephritis. The gastrointestinal mucosa was also characterized by marked necrosis and disintegration, with large numbers of bacteria. The heart displayed severe myocarditis and the lung displayed severe necrosis. During the experiment, there were no obvious histopathological changes in the muscles and mild inflammation and muscle-fiber necrosis were only observed in the high-concentration groups (1 × 10^9^ and 1 × 10^10^) (Figure 3(a)).

We also observed ultrastructural pathological changes in *E. miricola*-infected *P. nigromaculatus* (Figure 3(c)), which were mainly caused by the swelling of the mitochondria and endoplasmic reticulum. Furthermore, *E. miricola* was detected both intracellularly and in the interstitium of *P. nigromaculatus.*

### 3.4. Organs Parasitized by *E. miricola* in *P. nigromaculatus*

**Figure 4:**
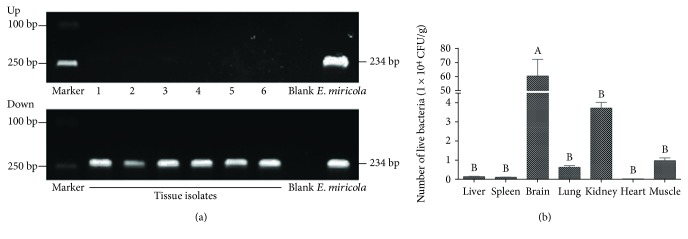
Organs parasitized by *E. miricola* in *P. nigromaculatus*. (a) PCR identification of bacteria in diseased frogs. Up: identification of *E. miricola* with 16S-rRNA-specific primers; 1: *Escherichia coli*; 2: *Klebsiella pneumoniae*; 3: *Aeromonas veronii*; 4: *A. hydrophila*; 5: *Pseudomonas plecoglossicida*; 6: *Stenotrophomonas* sp. Down: confirmation of strains of *P. nigromaculatus* in the experimental group. (b) Tissue distribution of *E. miricola* in *P. nigromaculatus*. Different lowercase letters indicate significant differences between groups.

To confirm that the lesions in *P. nigromaculatus* were caused by *E. miricola*, a PCR assay was conducted with primers specific for the 16S rRNA of *E. miricola* (Figure 4(a)). When bacteria enter the host, they usually target the organ that best satisfies the requirements for their maintenance and proliferation. Colony counts indicated that the brain was the principal target organ of *E. miricola* in *P. nigromaculatus*, because it contained very large amounts of parasitic bacteria. The kidney was the second most important target organ of *E. miricola* and contained only ~16 times fewer bacteria than the brain. The bacterial contents in the remaining tissues were much lower than in these two organs (Figure 4(b)). The result indicates that the brain is the favorite parasitic target organ of *E. miricola*, which may be the cause of meningoencephalitis.

### 3.5. *Elizabethkingia miricola* Inhibits the Antioxidant Capacity of *P. nigromaculatus*

**Figure 5:**
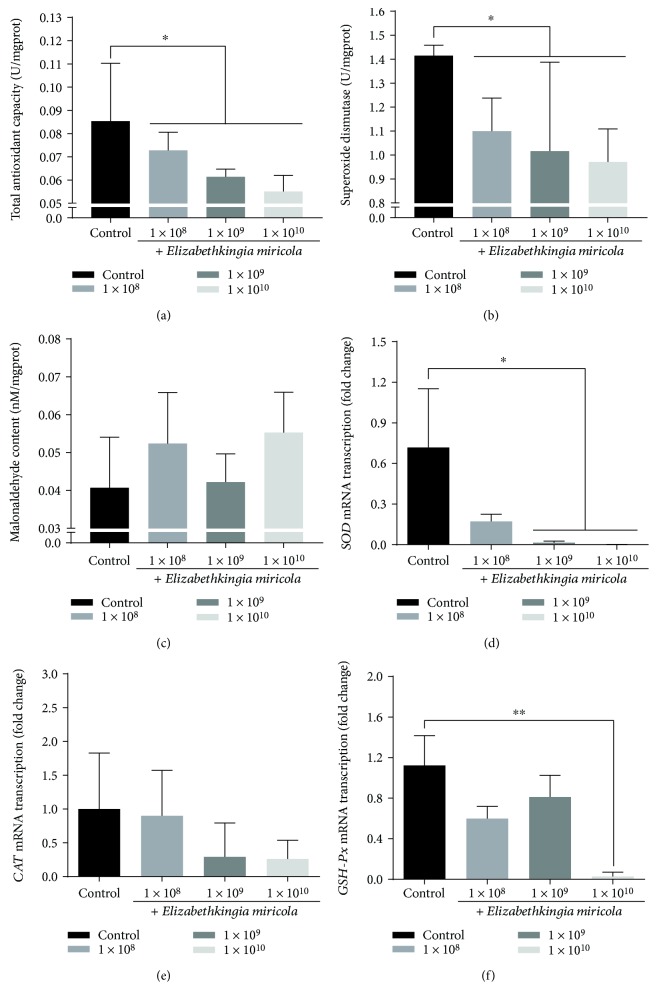
Antioxidation capacity of *P. nigromaculatus* in different groups. (a–c) Assessment of TAC, SOD, and MDA in *P. nigromaculatus* in different groups. (d–f) Expression of *SOD*, *CAT*, and *GPX* mRNAs in *P. nigromaculatus*. *P* < 0.05 or ^∗∗^*P* < 0 01 represents a significant difference or highly significant difference between groups.

The antioxidant system plays an important role in eliminating environmental and disease-induced oxidative stress. To explain the observed swelling of the mitochondria and the ER, we determined the antioxidant capacity of *P. nigromaculatus* after infection with *E. miricola.* Our results showed that *E. miricola* caused a significant reduction in TAC activity in the liver of *P. nigromaculatus*, and as the concentration of *E. miricola* increased, this reduction became more pronounced (Figure 5(a)). When the activity of SOD alone was measured, it was also significantly lower after *E. miricola* infection than in the control group (Figure 5(b)). These results indicate that *E. miricol*a weakens the antioxidant capacity of the *P. nigromaculatus* liver.

Reduced antioxidant capacity may lead to lipid peroxidation in the frog and cause the deposition of MDA. It is noteworthy that *E. miricola* infection increased the MDA content of *P. nigromaculatus*, although this increase was not statistically significant (Figure 5(c)).

The transcription of three genes associated with antioxidation (*SOD*, *CAT*, and *GPX*) was also measured. All three genes showed reduced expression after *E. miricola* challenge, indicating a disordered antioxidation system. Among these genes, *SOD* transcription was significantly reduced in the 1 × 10^9^ and 1 × 10^10^ groups and *GSH-Px* transcription in the 1 × 10^10^ group relative to the control group (Figures 5(d) and 5(f)). However, *CAT* transcription did not differ significantly between the control and experimental groups (Figure 5(e)).

## 4. Discussion

**Figure 6:**
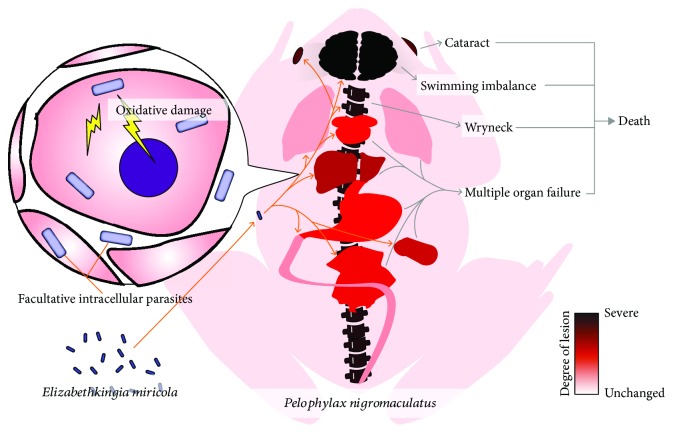
Systemic infection model of *E. miricola* against *P. nigromaculatus.*

*Elizabethkingia* is a new genus of bacteria, and most of its species are highly pathogenic to immunocompromised humans and animals [6–8]. Among these species, some subtypes of *E. meningoseptica*, *E. miricola*, and *E. anopheles* can cause meningitis in humans or animals and cause incurable disease and death in large numbers of patients [6, 14]. The isolation of *E. miricola* from *P. nigromaculatus* with wryneck disease in the present study has undoubtedly extended our understanding of the pathogenic spectrum of the genus *Elizabethkingia*. Because the lesions caused by *E. miricola* in *P. nigromaculatus* were similar to those caused by other *Elizabethkingia* spp. in humans, *P. nigromaculatus* may have utility as a model organism in the prevention and treatment of *Elizabethkingia-*induced human diseases. In this study, the systematic pathology of and oxidative damage caused by *E. miricola* in *P. nigromaculatus* were investigated with retrogressive infection in the laboratory. Our results show that *E. miricola* is a bacterium that mainly parasitizes the host brain and that the neurogenic organs are its main targets. The main damage caused was severe bacterial necrotic inflammation. The dysregulation of the *P. nigromaculatus* antioxidant system during infection may have aggravated these pathological processes (Figure 6). Our results provide a preliminary reference for the study and treatment of *Elizabethkingia-*induced neurological diseases.

In this study, we found that the brain is the primary target organ of *E. miricola* and *P. nigromaculatus* showed typical neurological symptoms, including wryneck, body bias, and cataract. Histopathology further clarified the clinical lesions caused by this bacterium, which mainly manifested as typical severe bacterial purulent meningoencephalitis, severe bacterial necrotic myelitis, and severe bacterial necrotic retinitis. Similar results have been demonstrated in humans. Previous studies have reported that *Elizabethkingia* spp. are often associated with meningitis and spondylitis in newborns and immunocompromised adults [30] and that *Elizabethkingia* spp. primarily affects neurogenic organs. However, these bacteria can also cause systemic infections. We found small numbers of bacteria distributed in the muscle, lung, spleen, and liver, with different degrees of histopathological disturbance. There was obvious bacterial necrotic inflammation in the liver, spleen, kidney, heart, and gastrointestinal tract. Unfortunately, we did not count the bacteria in the gastrointestinal tract to avoid bacterial contamination. However, the gastrointestinal tract was one of the main target organs, showing large numbers of rod-shaped bacteria in the lamina propria and submucosa, accompanied by severe, histopathologically detected, bacterial necrotic gastroenteritis. These findings indicate that infection with *E. miricola* causes multiorgan damage in *P. nigromaculatus* and that these symptoms are quite similar to those observed during the infection of humans by *Elizabethkingia* spp. *Elizabethkingia* spp. reportedly causing a variety of diseases, including pneumonia, bacteremia, sepsis, endocarditis, abdominal abscess, sinusitis, bronchitis, epididymitis, and dialysis-related peritonitis in immunocompromised adults [31] and bacterial necrotic inflammation may be a common pathological condition caused by *Elizabethkingia* spp.

The antioxidant system plays an important role in eliminating environmental and disease-induced oxidative stress [32]. An outbreak of disease often disrupts the antioxidant system, generates numerous free radicals, and causes oxidative damage to the body. The swelling of the mitochondria and ER suggests that *E. miricola* causes oxidative stress in *P. nigromaculatus* [33], which often results in mitochondrial stress and the unfolded protein response and in turn to removes intracellular free radicals [34]. The detection of antioxidants and the increased expression of antioxidation-associated genes indicated that the antioxidant capacity of *P. nigromaculatus* was compromised during *E. miricola* infection. These results indicate that an animal may gradually lose its antioxidant system after it is infected by *Elizabethkingia* spp., which may further aggravate its disease state.

Previous studies have reported that *Elizabethkingia* spp., which are Gram-negative bacteria, show excellent resistance to antibiotics, which may explain why patients are difficult to cure of these infections [35–37]. We found that *E. miricola* mainly parasitizes the neurological organs, such as the brain. It can live outside the intercellular matrix, and a few bacteria may be able to enter the cytoplasm. Most intracellular bacteria, such as *Staphylococcus aureus* [38] and *Mycobacterium tuberculosis* [39], are difficult to treat with drugs because they are protected by the cell membrane. This may also explain why the drug treatment of the human meningitis caused by *Elizabethkingia* spp. infection is ineffective. Although some antibiotics may be highly toxic to these bacteria, their poor liposolubility may inhibit their entry into the cell [40]. Therefore, drugs that are more liposoluble and that are more ready to penetrate the blood-brain barrier should offer more effective therapeutic options for *Elizabethkingia* spp.-induced diseases.

## 5. Conclusions

Genus *Elizabethkingia* is a new type of human opportunistic pathogen, commonly infecting newborns and immunocompromised adults, leading to meningitis, brain abscess, and many other conditions. Cases infected with *Elizabethkingia* usually lead to more than 50% mortality, causing serious public safety problems. This study established the *E. miricola* infection of the *P. nigromaculatus* model, to understand the pathological damage of *Elizabethkingia* to animals. We found that *E. miricola* is a bacterium that could be facultative intracellularly parasitic in the host, which could cause systemic damage to the *P. nigromaculatus* by producing oxidative stress. *E. miricola* prefers to be parasitic in nerve tissue, causing significant neuronal necroinflammation in the brain, spine, and eyeballs (Figure 6). The present study helps to further understand the damage to the host of *Elizabethkingia* spp. Also, *P. nigromaculatus* may become a candidate animal disease model of *Elizabethkingia* spp. infection in humans.

## Figures and Tables

**Table 1:** Histopathological assessment. An importance factor (*ω*_IF_), ranging from 1 to 3, was assigned to every change (1—minimal pathological importance: the lesion is easily reversible as exposure to irritants ends; 2—moderate pathological importance: the lesion is reversible in most cases if the stressor is neutralized; and 3—marked pathological importance: the lesion is generally irreversible, leading to partial or total loss of the organ function [26]).

Organ (Org)	Functional unit of the tissue (*t*)	Alteration (alt)	Importance factor (*ω*_IF_)
Kidney	Tubule/glomerulus/interstitial tissue		
Liver	Liver tissue/interstitial tissue	Haemorrhage	1
Heart	Epicardial/myocardium/cardiac chamber	Hyperaemia	
Lung	Alveolar/capillaries	Edema	1
Spleen	Interstitial tissue/sinusoid	Deposition	1
Muscle	Muscle fiber/connective tissue	Hypertrophy	1
Brain	Meninges/brain parenchyma	Hyperplasia	2
Spine	Nerve tissue/muscle tissue/connective tissue	Atrophy	2
Eye	Retina/cornea	Infiltration	2
Stomach	Mucosa/smooth muscle layer	Necrosis	3
Intestinal	Mucosa/lamina propria/smooth muscle layer		

**Table 2:** Primers for the variety of genes detected with qPCR.

Gene	Abbreviation	Primer sequence (5′–3′)	Acc. number
*Superoxide dismutase 2*	*SOD2*	F: AACCTGAATATTGCAGAGGAGAAGTAC	XM_018565611.1
		R: GCAATCTGAGCTGTAACATCTCCTT	

*Catalase*	*CAT*	F: ATTTCTGGGCTCTGCGTC	MK561295
		R: GGTTCATCCTTGGCGTTA	

*Glutathione peroxidase*	*GSH-Px*	F: TGCCGCTGTTCACCTTCCT	MK561296
		R: AAGTTCCAGGAGATGTCGTTGC	

*Ribosomal protein L8*	*rpl8*	F: GCTGTCGACTTCGCAGAAAGGCA	XM_018556352.1
		R: ACCTGTAAGGGTCACGGAAGGCA	

*18S ribosomal RNA*	*RNA 18S*	F: CGTTGATTAAGTCCCTGCCCTT	AB099628.1
		R: GCCGATCCGAGGACCTCACTA	

**Table 3:** Biochemical characteristics of the isolated strains.

Item	Fy70815 1-3	*E. meningoseptica* [2]	*E. miricola* [3]	*C. taichungense* [3]
Gram staining	-	-	ND	ND
Mobility	-	-	ND	ND
Catalase	+	+	ND	ND
Xylose	-	-	-	(+)
Fructose	+	+	+	ND
Esculin	+	+	+	+
Indole	N	N	V	(+)
Hydrogen sulfide	-	-	V	-
Nitrate	-	-	-	-
Ornithine decarboxylase	-	-	ND	ND
Lysine decarboxylase	-	-	ND	ND
Arginine decarboxylase	-	ND	ND	ND
Arginine double hydrolysis	N	-	ND	ND
Urease	V	-	ND	ND
V-P test	-	-	ND	ND
Arabinose	N	N	-	-

+: positive reaction; -: negative reaction; N: not applicable; V: variable reaction; (+): weak or delayed reaction; ND: not determined.

**Table 4:** Sensitivity of the three isolates to antibiotics.

Antibiotic	Standard (mm)			Inhibition zone (mm)	Sensitivity range
	Resistant (R)	Moderate (I)	Sensitive (S)		
Azithromycin	≤13	14—17	≥18	20	S
Enrofloxacin	≤14	15—17	≥18	15	I
Neomycin	≤12	13—16	≥17	11	R
Doxycycline	≤12	13—15	≥16	6	R
Penicillin	≤19	20—22	≥23	6	R
Rifampicin	≤16	17—19	≥20	20	S
Compound neoproxine	≤10	10—16	≥16	7	R
Cefoxitin	≤14	15—17	≥18	6	R
Ampicillin	≤14	15—19	≥20	6	R
Florfenicol	≤12	13—17	≥18	25	S

R: resistant; I: moderately sensitive; S: sensitive.

## Data Availability

The data used to support the findings of this study are available from the corresponding author upon request.
